# Acceptance of Treatment of Sexually Transmitted Infections for Stable Sexual Partners by Female Sex Workers in Kampala, Uganda

**DOI:** 10.1371/journal.pone.0155383

**Published:** 2016-05-12

**Authors:** Yunia Mayanja, Aggrey David Mukose, Susan Nakubulwa, Gloria Omosa-Manyonyi, Anatoli Kamali, David Guwatudde

**Affiliations:** 1 MRC/UVRI Uganda Research Unit on AIDS, Entebbe, Uganda; 2 Department of Epidemiology and Biostatistics, School of Public Health, College of Health Sciences, Makerere University, Kampala, Uganda; 3 College of Health Sciences, School of Medicine, University of Nairobi, Nairobi, Kenya; Simon Fraser University, CANADA

## Abstract

**Background:**

The prevalence of sexually transmitted infections (STIs) among female sex workers (FSWs) in sub-Saharan Africa remains high. Providing treatment to the affected FSWs is a challenge, and more so to their stable sexual partners. There is scanty research information on acceptance of STI treatment for stable sexual partners by FSWs. We conducted a study to assess acceptance of STI treatment for stable sexual partners by FSWs, and to identify factors associated with acceptance.

**Methods:**

We enrolled 241 FSWs in a cross sectional study; they were aged ≥ 18 years, had a stable sexual partner and a diagnosis of STI. Factors associated with acceptance of STI treatment for stable sexual partners were analysed in STATA (12) using Poisson regression. Mantel-Haenszel tests for interaction were performed.

**Results:**

Acceptance of partner treatment was 50.6%. Majority (83.8%) of partners at the last sexual act were stable partners, and 32.4% of participants had asymptomatic STIs. Factors independently associated with acceptance were: earning ≤ $4 USD per sexual act (aPR 0.68; 95% CI: 0.49–0.94) and a clinical STI diagnosis (aPR 1.95; 95% CI: 1.30–2.92). The effect of low income on acceptance of partner treatment was seen in those with less education.

**Conclusion:**

Acceptance of STI treatment for stable sexual partners was lower than that seen in other studies. Interventions to improve economic empowerment among FSWs may increase acceptance of partner treatment.

## Introduction

Sub-Saharan Africa is one of the regions most affected by the four main curable sexually transmitted infections (STIs) which are gonorrhoea, chlamydia, syphilis and trichomoniasis [1]. The highest STI prevalence has been reported among key populations such as female sex workers (FSWs) [2–4] and fisher folk [5, 6]. The reported STI prevalence among Ugandan FSWs is 13% gonorrhoea, 9% chlamydia, 10% syphilis and 17% trichomoniasis [4]. STI control interventions that target both individuals and communities [7–9] are necessary to prevent STI transmission and complications, and reduce HIV acquisition and transmission. They comprise of structural interventions that provide an enabling environment, behavioural interventions such as behaviour change communication and condom programming, working synergistically with biomedical interventions. Periodic presumptive treatment is a biomedical intervention that has been recommended for short term use among FSWs [10]; others are syndromic case management, screening for and treatment of asymptomatic infections, managing STIs in HIV positive individuals and partner notification [11]. Partner notification (PN) is important in STI management and yet not widely studied in sub-Saharan Africa [12], more so among FSWs who report to have stable sexual partners. PN can be implemented either through partner referral or through the index patient taking medication or a prescription to their sexual partner(s) [13–16]. Although the practice of delivering drugs to partners is not widely implemented globally, studies have shown that 74–80% of index STI patients take treatment for their sexual partners [13–16]. Randomised trials have shown this method to reduce re-infection by 27% more when compared to standard partner referral [17] and to increase the number of sexual partners treated by 19–40% [15, 18, 19]. It has also been found to be more cost effective than standard partner referral [20, 21].

In Uganda, like in many sub-Saharan countries, sex work is illegal and in some cases FSWs encounter challenges such as poor attitudes from health workers [22, 23], police brutality and stigmatisation from their communities [22]. Studies show that some Ugandan FSWs have disadvantaged backgrounds characterised by parental neglect, experiences of violence in childhood and limited access to economic resources [22, 24]. Most of the women are young, separated from their spouses and have low education levels [4, 25]; they therefore lack the knowledge or skills to find alternative well-paying jobs, and the need to earn money for child care is the main reason given for remaining in sex work [25]. The sexual relationships of FSWs involve stable partners who may be spouses (non-paying partners) and/or regular paying clients, and casual paying partners. FSWs sometimes accept regular clients to have sex with them then pay later. The women have their own social structure as follows: those who have no capital and depend entirely on sex work for their livelihood, those working in entertainment facilities and engage in a more institutionalised sex trade often mediated by middlemen, and the minority who are more financially independent women and earn from their own businesses as well as from sex work [24]. Violence against FSWs is common and is perpetrated by both clients and police [22, 26]. A high prevalence of both physical and sexual violence (82%) has been reported and increases with increasing demands for unprotected sex by clients [27]. Violence is reported to be higher among street based FSWs [26]. Although the indoor locations such as bars are safer, FSWs are still exploited by managers and middlemen who extort money from them [22]. The risk of violence among these women is considered to be far greater and more immediate than HIV [22], and yet they are disempowered by lack of a structure where they can seek legal redress to these violations. Furthermore, FSWs have reported feeling powerless in bargaining for and maintaining condom use which is usually dependent on client willingness, price offered and the woman’s sobriety [26, 28]. Reported condom use between stable sexual partners and FSWs in Uganda has been documented to be low (6%) compared to 60% with casual partners [4]; hence stable sexual partners of FSWs need to be targeted for treatment. Sexual partners of FSWs should also be targeted by interventions aimed at improving gender equality, power relations and partner communication. Male involvement is important in promoting safer sexual practices and empowering women to embrace economic and social opportunities for better health [25].

FSWs have limited access to sexual and reproductive health and rights (SRHR) interventions which include but are not limited to: sexuality education, access to reproductive and psychosocial health services without discrimination, coercion or violence and the right to sexual freedom, equity, privacy and autonomy. The criminalisation of sex work on the other hand perpetuates acts of cruelty, humiliation and sexual abuse from the public and authorities [22, 26], and also hinders efforts to prevent HIV and other STIs among FSWs. Despite a high STI prevalence among FSWs [2, 3], there is limited knowledge on interventions for partner treatment among this key population in Uganda and other parts of sub-Saharan Africa. We studied acceptance of STI treatment for stable sexual partners by FSWs and factors associated with acceptance.

## Methods

### Study design, setting and participants

We performed a cross sectional study nested within a cohort of 1027 FSWs. The women were either self-identified sex workers or employed in entertainment facilities and were engaging in sexual intercourse with a man other than their spouse, in exchange for money, goods or favours. The cohort was established at the Good Health for Women project clinic, in a peri-urban community in southern Kampala between 2008 and 2009 with the aim of studying the epidemiology of HIV and other STIs, and offering a platform for future HIV prevention intervention trials. Vandepitte has described recruitment of the cohort from commercial hotspots [4]. Participants gave written informed consent to take part in studies on the epidemiology of HIV and other STIs, and gave contact details; they attended an enrolment visit where study staff collected research data on socio-demographic variables, sexual behaviour, alcohol and drug use, contraception, pregnancy and genital symptoms. Every participant gave blood and genital samples for laboratory STI diagnosis whether they had symptoms or not. The study staff provided HIV counseling and testing, screening and treatment for STIs, general health care, family planning services and free male condoms. Symptomatic participants with vaginal discharge syndrome (VDS), pelvic inflammatory disease (PID) or genital ulcer disease (GUD) were treated using the syndromic approach as written in the Uganda Clinical Guidelines that are derived from WHO guidelines [29]. The asymptomatic study participants were called back to the clinic for treatment if laboratory tests showed an infection; those who had been treated clinically were not called back unless laboratory tests revealed an organism(s) that does not cause the syndrome they were treated for. After enrolment, participants were asked to attend quarterly follow up visits where study procedures were similar to enrolment and contact details updated. The study staff referred those confirmed to be HIV positive to a facility that provided anti-retroviral therapy (ART) when they became eligible for HIV treatment. Study staff also provided counselling to the cohort participants on partner notification, treatment compliance and condom use whenever they were diagnosed with STIs. Participants were encouraged to refer sexual partners to general health facilities of their choice for treatment, but we did not document if this happened. It was logistically difficult to invite the stable sexual partners to a clinic designated for FSWs. The practice of offering FSWs STI drugs for stable sexual partners was implemented in the cohort in 2010 (24 months after enrolling the first participant); treatment was offered using STI diagnosis of the woman. We selected our study participants from those who attended month 24 in the cohort (April 2010 to May 2011) so as to include participants with the same follow up duration. At the time of their month 24 visit, most (76.5%) of cohort participants with STIs had never been offered STI treatment for partners at the study clinic, and periodic presumptive treatment was not yet a recommended guideline for STI control among FSWs.

Eligible participants were consecutively enrolled into the study until a sample size of 241 was reached. We included participants aged 18 years or more who reported having a stable sexual partner and were diagnosed with STI(s). A stable sexual partner was defined as one with whom a woman had been having a continuous sexual relationship for at least three months preceding the month 24 visit, with the intention of continuing that relationship; a casual partner was one with whom a woman had a one-off or occasional paid sexual encounters for at most three months before the month 24 visit with no intention of a long lasting relationship. STIs considered for inclusion were: a clinical diagnosis of vaginal discharge syndrome (VDS) and pelvic inflammatory disease (PID); and laboratory diagnosis of gonorrhoea, chlamydia, syphilis, chancroid and trichomoniasis. We excluded participants with a laboratory diagnosis of herpes simplex type 2 (HSV-2); vaginal candidiasis and/or bacterial vaginosis (BV); and a clinical diagnosis of genital ulcer disease (GUD) (because we previously showed no etiology in 55% of genital ulcers [4]).

We offered treatment to the participants to deliver to their stable sexual partners; in cases where the participant had multiple stable partners, she selected the one to give treatment. Limited resources permitted STI treatment for only one stable sexual partner per participant. Acceptance of partner treatment was defined as a FSW diagnosed with an STI(s) picking STI drugs for her stable sexual partner from the clinic pharmacy. We did not verify whether or not treatment was delivered to the stable sexual partner.

### Sample size

At 5% level of significance, we estimated that a sample size of 196 would give 80% power to detect a significant difference of at least 9% in acceptance of partner treatment among participants, our assumption is based on 74% as the proportion of STI patients who took partner treatment in a Ugandan study conducted in the general population [15]. The proportion of asymptomatic STIs reported from this cohort is 58% [4]. Due to estimated loss to follow up of 24% for asymptomatic participants called back to the clinic after laboratory results tested positive (estimated from Huppert’s study) [30], the overall sample size was increased to 224 participants.

### Laboratory methods

At month 24 visit the following samples were taken: an endo-cervical swab for diagnosis of *Neisseria gonorrhoea* and *Chlamydia trachomatis*, using the Amplicor CT/NG PCR test (Roche diagnostic Systems Inc., Branchburg, NJ); three vaginal swabs for: gram staining to determine the Nugent score for *Bacterial vaginosis*, wet mount slide for microscopic detection of *Trichomonas vaginalis* and *Candida albicans* and inoculation for culturing *T*. *vaginalis* (InPouch TV, Biomed Diagnostics, San Jose, CA, USA); Dacron swab from genital ulcers to diagnose *Haemophilus ducreyi*, *Treponema pallidum*, *HSV-1&-2* using a multiplex Real Time PCR assay (Roche Light Cycler) and 5mls of blood for syphilis serology tests, and HIV testing.

Syphilis serology was done using a quantitative Biotec RPR and the *Treponema Pallidum* HaemAgglutination Assay (TPHA). Positive syphilis serology was RPR titres ≥ 1:8, any titre values in previously RPR negative participants and increase in titres from the previous test. HIV testing was performed using a highly sensitive rapid test (Abbott Determine). Non-reactive tests were considered HIV negative. Reactive or indeterminate results were confirmed by two enzyme linked immunosorbent assay (ELISA) tests performed in parallel (Vironostika Uniform II plus O, Murex HIV 1.2.O). If both ELISA tests were positive, then HIV result was reported as positive. If results were discordant or equivocal, a Western Blot Test (Cambridge Calypte Western Blot) was performed to resolve the status.

### Study variables

The study outcome was acceptance of partner treatment by the study participant, a dichotomous variable estimated as a proportion. Acceptance was taken as “yes” if participants with symptomatic STI(s) took drugs for partners after diagnosis using syndromic approach and if asymptomatic participants took drugs after laboratory diagnosis.

Independent variables included: condom use between FSWs and partner at the last sexual act (Yes/No), time between last sexual act and month 24 visit (≤ 1 week / > 1 week) and total number of sexual partners in the past three months. Covariates included: age at enrolment in the cohort (continuous variable); education level, income earned per sexual act in the past month (continuous variable), marital status, main occupation (sex work/ sex work and other job), religion, HIV status, type of partner at last sexual act, length of relationship with partner at last sexual act, and presence of STI symptoms at month 24 visit. All variables were collected using questionnaires.

### Statistical Methods

We performed data analysis using Stata 12 (StataCorp, College Station, Texas 77845 USA). All independent variables and covariates were analysed as categorical variables using Poisson regression with robust error variance to give prevalence ratios (PR). Since there was collinearity between “length of relationship with partner at the last sexual act”, “type of partner at the last sexual act” and “condom use between participants and partner at the last sexual act”; we retained the latter variable in the final analysis. We constructed a multivariable model using a hierarchical framework based on three levels: biological factors, sexual behavioural factors and socio-demographic variables. Models were constructed for each level by retaining variables that were significant (p ≤ 0.05) at univariate analysis. The significant variables at univariate analysis were then fitted individually and in groups within each level; the best model for each level was the one with the lowest value for the Akaike information criteria (AIC). We then constructed the adjusted model based on significant relationships that we observed in our data by including significant variables from each level based on p- value (<0.05) and AIC. The variables “number of sexual partners in the past three months” [31, 32] and “type of partner at the last sexual act” [13] were added a priori based on literature. Mantel-Haenszel chi squared test was used to test for interaction between independent variables in relation to the outcome variable. Results are presented as prevalence ratios (PR) and adjusted prevalence ratios (aPR) with the corresponding 95% Confidence Intervals (CI).

### Ethical considerations

Uganda National Council for Science and Technology (SS 3107) and Uganda Virus Research Institute-Research Ethics Committee (GC/127/13/03/29) gave written ethical approval for our study. Written informed consent for studies on HIV and STIs had been obtained from all participants in the parent cohort, and it allowed this analysis. We kept confidentiality by use of numerical identifiers during data collection. Before analysis, all data including numerical identifiers were de-identified.

## Results

### Study participants

**Fig 1:**
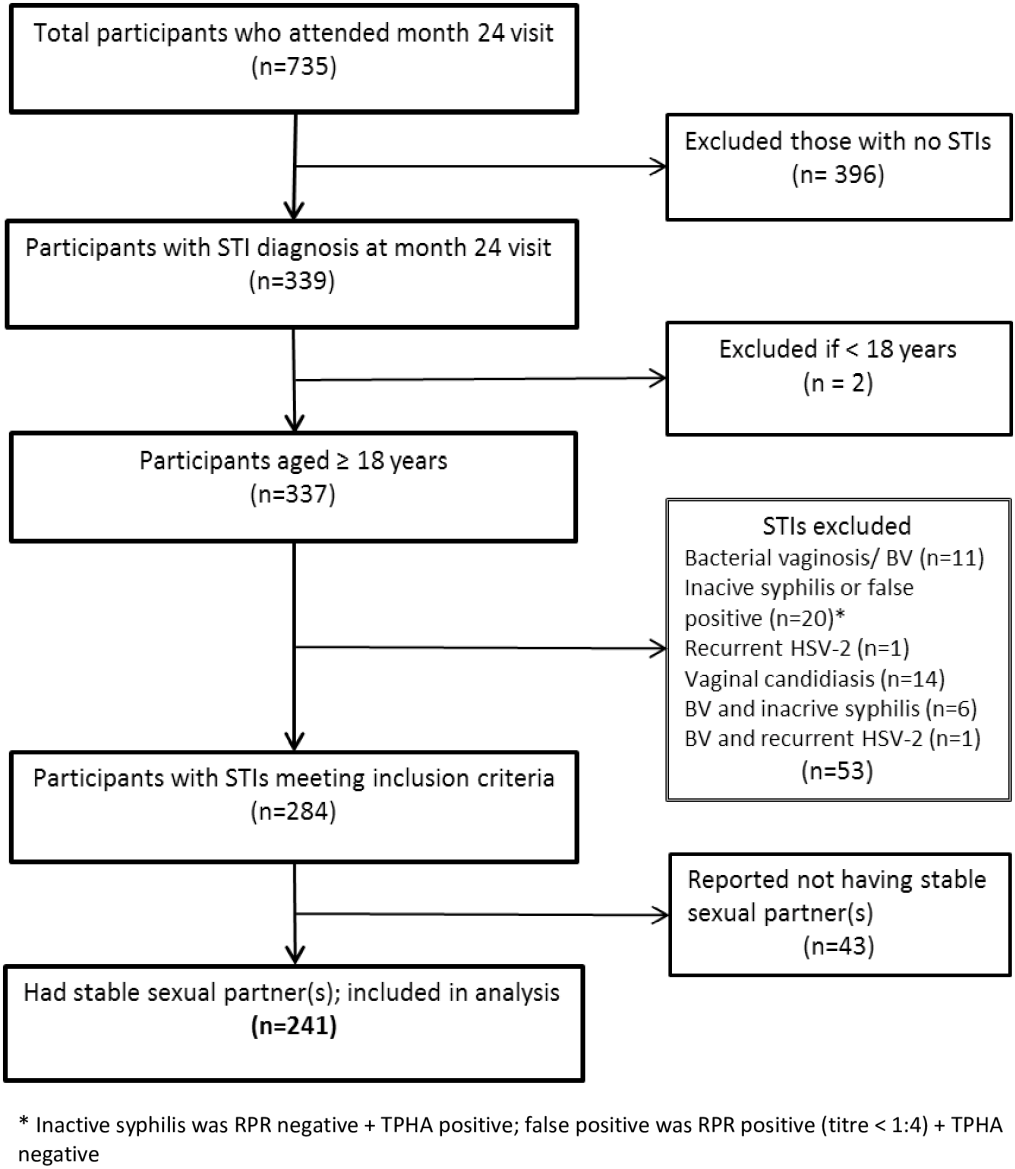
Screening profile of women attending month 24 clinic visit in the Good health for Women project cohort.

Overall, 735 women attended their month 24 visit in the main cohort between April 2010 and May 2011, 494 did not meet eligibility criteria and were excluded from our study due to the following: 396 did not have an STI, 53 did not have a treatable or notifiable STI, 43 had no stable sexual partners, and 2 were aged below 18 years. We enrolled a total of 241 participants in our analysis. The screening schema is given in Fig 1.

### Socio-demographic characteristics of study participants

The mean (±SD) age of the 241 enrolled participants was 26 ± 5 years. A half of the participants (50.6%) had primary level education, 62.2% had ever been married, and 73.9% were Christians. The median amount of money earned per sexual act was $ 3.2 USD (IQR 2.00–8.00), and sex work was the sole source of income for 41.5% of the participants. Of 141 who had other income, 94 (66.7%) worked in a bar, guest house or massage parlour. Details are shown in Table 1.

**Table 1:** Socio-demographic Characteristics of Study Participants.

Characteristic	Categories	Frequency (n = 241)	Percentage (%)
**Age at enrolment (years)**			
	18–24	109	45.2
	25–29	78	32.4
	30–34	39	16.2
	≥ 35	15	6.2
**Education level**			
	None	25	10.4
	Primary	122	50.6
	Secondary or higher	94	39.0
**Current marital status**			
	Never married	27	11.2
	Married/ cohabiting	64	26.6
	Widowed/ separated	150	62.2
**Ethnicity**			
	Baganda	140	58.1
	Other Ugandan	90	37.3
	Non-Ugandan	11	4.6
**Religion**			
	Christian	178	73.9
	Muslim	63	26.1
**Main occupation**			
	Sex work alone	100	41.5
	Sex work and other job	141	58.5
Income per sexual act (USD)*			
	No income	42	17.4
	0.80–4.00	138	57.3
	4.01–10.00	36	14.9
	> 10.00	25	10.4

* 1USD = 2,500 Uganda shillings during the study period.

### Sexual behaviour of study participants and occurrence of STIs

One hundred seventy three participants (71.8%) had a relationship lasting at least 6 months with the partner at the last sexual act, 103 (42.7%) reported condom use with partners at the last sexual act, 145 (60.2%) had the last sexual act within less than a week prior, and 138 (57.3%) had more than one sexual partner in the past 3 months. Majority of the participants (83.8%) reported to have had the last sexual act with a stable partner. Condom use between participants and the partner at the last sexual act was 32.2% (65/202) with stable partners compared to 97.4% (38/39) with casual partners. Symptomatic STIs were diagnosed in 163 (67.6%) participants; of 78 who did not have symptoms, 16 (20.5%) had chlamydia, 12 (15.4%) gonorrhoea, 25 (32.1%) syphilis, 19 (24.4%) trichomoniasis and the rest had multiple infections. One hundred eleven (41.6%) participants were HIV positive. Details are shown in Table 2.

**Table 2:** Sexual Behaviour and STIs among Study Participants.

Characteristic	Categories	Frequency (n = 241)	Percentage (%)
**Condom use between FSWs and partners at last sexual act**			
	Yes	103	42.7
	No	138	57.3
**Time between last sexual act and month 24 visit**			
	< 1 week	145	60.2
	≥ 1 week	96	39.8
**Type of partner at last sexual contact**			
	Stable partner	202	83.8
	Casual partner	39	16.2
**Relationship with partner at last sexual act**			
	No relationship	39	16.2
	< 6 months	29	12.0
	6–12 months	27	11. 2
	> 12 months	146	60.6
**Number of sexual partners in past 3 months**			
	None	8	3.3
	One	95	39.4
	2–9	51	21.2
	≥ 10	87	36.1
**Symptomatic STIs at month 24**			
	Yes	163	67.6
	No	78	32.4
**HIV status**			
	Positive	111	46.1
	Negative	130	53.9

### Acceptance of STI treatment for stable sexual partners and associated factors

A total of 122 (50.6%) participants accepted STI treatment for stable sexual partners. Acceptance of partner treatment was higher among participants who reported lack of condom use between oneself and the partner at the last sexual act (PR 1.32; 95% CI: 1.01–1.73), those who reported a shorter duration (<1 week) since the last sexual act (PR 1.35; 95% CI: 1.03–1.79) and those with clinically diagnosed STIs (PR 1.85; 95% CI: 1.31–2.63). Earning ≤ 4USD per sexual act and being widowed or separated were associated with lower acceptance of partner treatment (PR 0.68; 95% CI: 0.51–0.90) and (PR 0.63; 95% CI: 0.45–0.87) respectively.

At adjusted analysis, lower income earned per sexual act was associated with lower acceptance of partner treatment (aPR 0.68; 95% CI: 0.49–0.94) while having a clinically diagnosed STI (aPR 1.95; 95% CI: 1.30–2.92) was associated with higher acceptance of partner treatment as shown in Table 3.

**Table 3:** Multivariable model for acceptance of STI Treatment for stable sexual partners.

Group	Variable	Categories	-n-	# accept partner treatment (%)	PR (95% CI)	Adjusted PR * (95% CI)
*Socio-demographic variables*	**Current marital status**					
		Never married	27	18 (66.7)	1	1
		Married/ Cohabiting	64	41 (64.1)	0.96 (0.69–1.33)	0.90 (0.60–1.36)
		Separated/ Divorced	150	63 (42. 0)	**0.63 (0.45–0.87)**	0.76 (0.55–1.05)
	**Income per sex act in USD (n = 199)** †					
		> 4.00 USD	61	37 (60.7)	1	1
		≤ 4.00 USD	138	57 (41. 3)	**0.68 (0.51–0.90)**	**0.68 (0.49–0.94)**
*Sexual behavioural variables*	**Condom use between FSWs and partners at last sexual act**					
		Yes	103	44 (42. 7)	1	1
		No	138	78 (56. 5)	**1.32 (1.01–1.73)**	1.22 (0.88–1.69)
	**Time between last sex act & month 24 visit**					
		≥ 1 week	96	40 (41.7)	1	1
		< 1 week	145	82 (56.6)	**1.35 (1.03–1.79)**	1.34 (0.96–1.86)
	**Number of sexual partners in past 3 months**					
		None	8	1 (12. 5)	1	1
		One	95	56 (58.9)	4.72 (0.75–29.83)	2.54 (0.47–13.80)
		2–9	51	25 (49.0)	3.92 (0.61–25.15)	2.62 (0.48–14.55)
		≥ 10	87	40 (45.9)	3.68 (0.58–23.42)	2.83 (0.51–15.65)
	**Type of partner at the last sexual act**					
		Regular	202	108 (53.5)	1	1
		Casual	39	14 (35.9)	0.67 (0.43–1.04)	0.78 (0.47–1.31)
*Biological variables*	**Clinical STI diagnosis at month 24 visit**					
		No	78	25 (32.1)	1	1
		Yes	163	97 (59.5)	**1.85 (1.31–2.63)**	**1.95 (1.30–2.92)**

* Adjusted for income per sex act, marital status, condom use between FSWs and partners at the last sexual act, duration since the last sexual act and clinical STI diagnosis.

^†^ 42 participants did not earn from sex work in the past one month.

### Stratified analysis for interaction

When stratified by education level and HIV status; participants with lower than secondary level education had lower acceptance for partner treatment if they earned ≤ 4USD per sexual act compared to those who earned more (PR 0.56; 95% CI 0.39–0.82); but there was no difference in acceptance of partner treatment by different income groups in those with higher education level (PR 0.88; 95% CI 0.57–1.36). HIV positive participants had higher acceptance of partner treatment if there was reported lack of condom use between oneself and the partner at the last sexual act compared to those who reported condom use (PR 1.74; 95% CI 1.12–2.69). This effect was not found among the HIV negative (PR 1.07; 95% 0.77–1.49).

## Discussion

We showed low acceptance of partner treatment among FSWs diagnosed with STIs; our study is among the few so far in sub Saharan Africa, and the first from Uganda to assess acceptance of partner treatment among FSWs. Studies that assessed patient delivered treatment found that 74% - 80% [13–16] of STI patients reported delivering STI treatment to sexual partners. One of the studies was a clinical trial done among high risk men but was stopped due to enrolment of insufficient numbers which affected reliability of the findings [14]. The other three studies were done among the general population in Brazil, Uganda and the United States [13, 15, 16]. Unlike the general population, it is possible that in our study setting where sex work is criminalised, FSWs may avoid talking about STIs with their stable sexual partners for fear that the clandestine nature of their occupation will be revealed to stable partners and other community members. The existing structural barriers and poor attitude towards women involved in sex work in Uganda and other parts of sub-Saharan Africa encourage stigmatisation, discrimination and violation of FSWs [22, 26]. This could contribute to low acceptance of partner treatment. Qualitative studies should define reasons for accepting or declining STI treatment for stable partners in this key population. Furthermore, a rights-based approach to strategies that build community support for FSWs is urgently needed and includes: legal reforms that address criminalisation and repressive policies against FSWS, access to health services that are acceptable to FSWs and avoid stigma and discrimination, partnerships with FSW-led organisations to address violence against FSWs and advocacy for better working conditions [10]. Interventions for FSWs have shown positive results when the wider community and policy makers are involved [33]. Without this enabling environment in Uganda and sub-Saharan Africa, STI control efforts for FSWs such as patient delivered treatment may fall short. Our study was limited by the fact that we provided treatment for one stable sexual partner yet some women had more than one stable sexual partner and could have declined treatment because they could not decide whom to give treatment. We also did not confirm if the stable partners received and took the treatment; we used non-random sampling methods and selected only those who attended their month 24 visit, which affects generalizability. The study participants had been attending quarterly research visits for two years and could have accepted to take partner treatment due to Hawthorne effect bias. Also, they had been receiving regular biomedical and behavioural interventions and may not be representative of all FSWs in Uganda and sub-Saharan Africa. Despite these limitations, our study highlights the difficult context for acceptance of patient delivered treatment for STIs by FSWs.

Overall FSWs with lower income per sexual act had lower acceptance of partner treatment. Having an income has been shown to be positively correlated with uptake of patient delivered treatment by STI patients elsewhere [34]. Our study participants generally had low income; this could have made them more vulnerable to violence from stable partners. Studies have shown that higher income empowers women and makes them less vulnerable to intimate partner violence (IPV) [35–37]. Additionally, among FSWs along the Trans-Africa highway in South-western Uganda, qualitative studies show that FSWs in the higher socio-economic class are empowered and therefore able to negotiate for higher pay and safer sex unlike those in the lower class [24]. The fear of IPV among FSWs with low income could prevent them from accepting to take STI treatment for stable partners. In neighbouring Kenya, a study has also shown an association between IPV and failure to deliver partner treatment for STIs [38]. There is a need to build FSW self-efficacy by providing opportunities for economic empowerment, not forgetting the importance of male partner involvement in empowering women to embrace strategies for better health [25], and preventing IPV.

We were not surprised by the higher acceptance of partner treatment among participants with a clinical STI diagnosis, and were treated using the syndromic approach. They received same day treatment and partner notification services unlike those who were asymptomatic and were called back for treatment days later when laboratory results were positive for STI(s). This explains the higher acceptance of partner treatment among those with a clinical STI diagnosis. Loss to follow up of STI participants who are called back to the clinic when laboratory results are available [30] also leads to missed opportunities for treating asymptomatic women and offering STI drugs for stable sexual partners. There was a high prevalence of asymptomatic STIs among our participants but these were missed by syndromic management which does not give a specific aetiological diagnosis [39]. Asymptomatic participants therefore did not receive same day treatment for themselves and their stable sexual partners. Other researchers have found that syndromic management is not suitable as a stand-alone method [40–42]. Furthermore, Mayaud and Mabey report that increased drug costs, alterations in vaginal flora, side-effects of multiple drugs and development of drug resistance are also likely limitations of the syndromic approach [43]. Although syndromic STI management is cost effective in resource limited settings, our findings indicate that there is need for regular laboratory screening for STIs among FSWs as has been reported before [4, 44]. However, available laboratory tests are expensive and do not give immediate results, leading to delay in treatment of asymptomatic women and offering partner treatment. Given the high STI prevalence among FSWS, periodic presumptive treatment is of value as a short term intervention[10], more novel, affordable and faster STI testing technologies are needed for the long term to enable timely treatment of FSWs and their stable sexual partners.

There exists valuable information from several partner notification approaches studied in various settings. Our study, being the first from Uganda to assess acceptance of partner treatment for stable sexual partners by FSWs not only adds to this knowledge but also provides a unique context among FSWs, who are an important group in STI control.

## Supporting Information

S1 Dataset (DTA)

S1 Checklist (DOCX)
